# Effects of traditional culture picture book intervention on emotional regulation ability and neuropsychology of preschool children

**DOI:** 10.3389/fpubh.2026.1832250

**Published:** 2026-05-08

**Authors:** Jing Wang

**Affiliations:** Tianjin Normal University Jingu College, Tianjin, China

**Keywords:** correlation, emotional regulation ability, intervention, preschool children, traditional culture picture books

## Abstract

The preschool stage is a critical period for children’s emotional regulation ability and cognitive function development. This study adopted a cluster randomized controlled trial design, selecting 120 4-6-year-old preschool children in Tianjin, who were randomly divided by class into an experimental group (*n* = 60) and a control group (*n* = 60). The experimental group received a 12-week traditional culture picture book intervention (twice a week), while the control group received regular kindergarten teaching without special picture book intervention. Both groups received equal class time and teacher attention to avoid confounding effects. The results showed that after the intervention, the experimental group performed significantly better than the control group in all indicators of emotional regulation ability and cognitive functions (all *p* < 0.001). Repeated-measures ANOVA revealed significant main effects of time, group, and time×group interaction (all *p* < 0.001). Pearson correlation analysis indicated a significant positive linear correlation between emotional regulation ability and cognitive functions (*r* = 0.426–0.578, *p* < 0.01). All results were reported with Cohen’s d effect sizes and 95% confidence intervals, and Bonferroni correction was applied for multiple testing. In conclusion, traditional culture picture book intervention can significantly improve preschool children’s emotional regulation ability and promote their cognitive function development, which can be incorporated into regular kindergarten teaching as an effective local intervention carrier. The correlation only demonstrates an association rather than a causal relationship between emotional regulation and cognitive functions.

## 1 Introduction

Preschool period (ages 4–6) serves as a critical window for brain development and psychological function formation in children. Emotional regulation and cognitive function development at this stage not only directly influence children’s current social adaptation, learning efficiency and mental health, but also lay a foundational role in long-term personality development and social functioning (1). Children in this period have an immature prefrontal cortex and highly plastic neural circuits. The neural mechanisms of emotional cognition, expression and control are developing rapidly. Meanwhile, children often show intense emotional responses, limited regulation strategies and easy generalization of negative emotions (2). Relevant epidemiological surveys show that about 20 to 40% of preschool children in China experience different levels of difficulties in emotional regulation. These difficulties include inaccurate emotional recognition, inappropriate expression and impulsive emotional reactions (3). Without scientific intervention, such problems may increase the risk of anxiety, attention deficit and other psychological and behavioral problems during school age (4).

Neuroscientific studies confirm that the development of emotional regulation in preschool children is closely related to cognitive functions, especially core cognitive functions such as executive function, attention and working memory (5). The prefrontal cortex acts as a common neural basis for emotional regulation and advanced cognitive functions. Its development directly affects key abilities including emotional response inhibition, cognitive reappraisal and attention allocation (6). For example, improved sustained attention helps children better perceive emotional signals from others, and greater working memory capacity supports the use of proper regulation strategies under emotional arousal (7). However, emotional education in early childhood education mainly relies on general activities. These activities are simple in form and lack cultural adaptation, making it difficult to stimulate children’s emotional resonance and active participation (8).

As an important carrier of cultural inheritance and educational practice, traditional culture picture books transform abstract emotional concepts and cultural connotations into understandable and experiential content for children through vivid stories, local cultural symbols and interesting expressions (9). Although culturally immersive education has become an important trend in preschool education, empirical studies on the systematic application of traditional culture picture books in interventions for emotional regulation and cognitive function development remain insufficient (10).

Most existing studies only evaluate intervention effects on a single dimension, such as changes in emotional cognition or behavior. They lack quantitative analysis of core cognitive function indicators, making it difficult to reveal the deep mechanisms of intervention (11, 12). In addition, localized evidence supporting the correlation between emotional regulation and cognitive functions is limited, which restricts the accurate understanding of the internal logic of intervention effects. There is a growing demand for localized intervention programs in preschool education. Traditional emotional education often shows unstable effects and poor sustainability due to insufficient cultural roots (13, 14). Traditional culture picture book intervention achieves the dual goals of cultural inheritance and psychological development. It is also easy to implement, cost-effective and compatible with routine kindergarten teaching. It meets the dual requirements of cultural confidence and quality-oriented education in current preschool education (15).

Therefore, targeted research on the effects of traditional culture picture book intervention on emotional regulation and cognitive functions in preschool children will help fill research gaps. It will also provide important theoretical and practical support for the construction of a localized and scientific intervention system for children’s psychological development.

## 2 Methods

### 2.1 Participants

This study selected preschool children aged 4–6 years from one public kindergarten in Tianjin using the convenience sampling method. A cluster randomized controlled trial was conducted with classes as the random unit to avoid intervention contamination. Only four classes were included (two experimental, two control), representing a small-sample cluster randomized design. The intraclass correlation coefficient (ICC) was pre-calculated as < 0.02, indicating extremely low clustering of individual observations within classes and negligible cluster effects that exerted no substantial impact on statistical power and result robustness. Therefore, cluster adjustment was not performed, which conforms to research specifications for small-sample cluster designs with extremely low ICC values (16, 17).

The inclusion criteria were as follows: (1) the age ranged from 4 to 6 years, with the month age between 48 and 72 months. (2) The children had no organic diseases or psychological and behavioral disorders that could affect emotional and cognitive assessment, such as intellectual disability, autism spectrum disorder, visual or hearing impairment. (3) Their parents provided informed consent and signed the informed consent form, and the children could cooperate in completing all assessment tasks. (4) The children had been studying in the kindergarten for at least 3 consecutive months to ensure an attendance rate of no less than 85% during the intervention. The exclusion criteria included three aspects: (1) participation in other specialized courses on emotional education or cognitive training during the intervention period. (2) Attrition due to relocation, kindergarten transfer or other reasons during the study process. (3) Incomplete assessment data that would affect statistical analysis.

A total of 120 children were finally enrolled and divided into the experimental group and the control group by class-based random number table method, with 60 children in each group. In the experimental group, there were 32 boys and 28 girls, with an average age of 5.23 ± 0.67 years, including 21 four-year-olds, 25 five-year-olds and 14 six-year-olds. In the control group, there were 31 boys and 29 girls, with an average age of 5.18 ± 0.72 years, including 23 four-year-olds, 24 five-year-olds and 13 six-year-olds. No statistically significant differences were found between the two groups in gender, age, baseline emotional regulation ability or cognitive function indicators (*p* > 0.05), indicating that the two groups were comparable.

**Figure 1:**
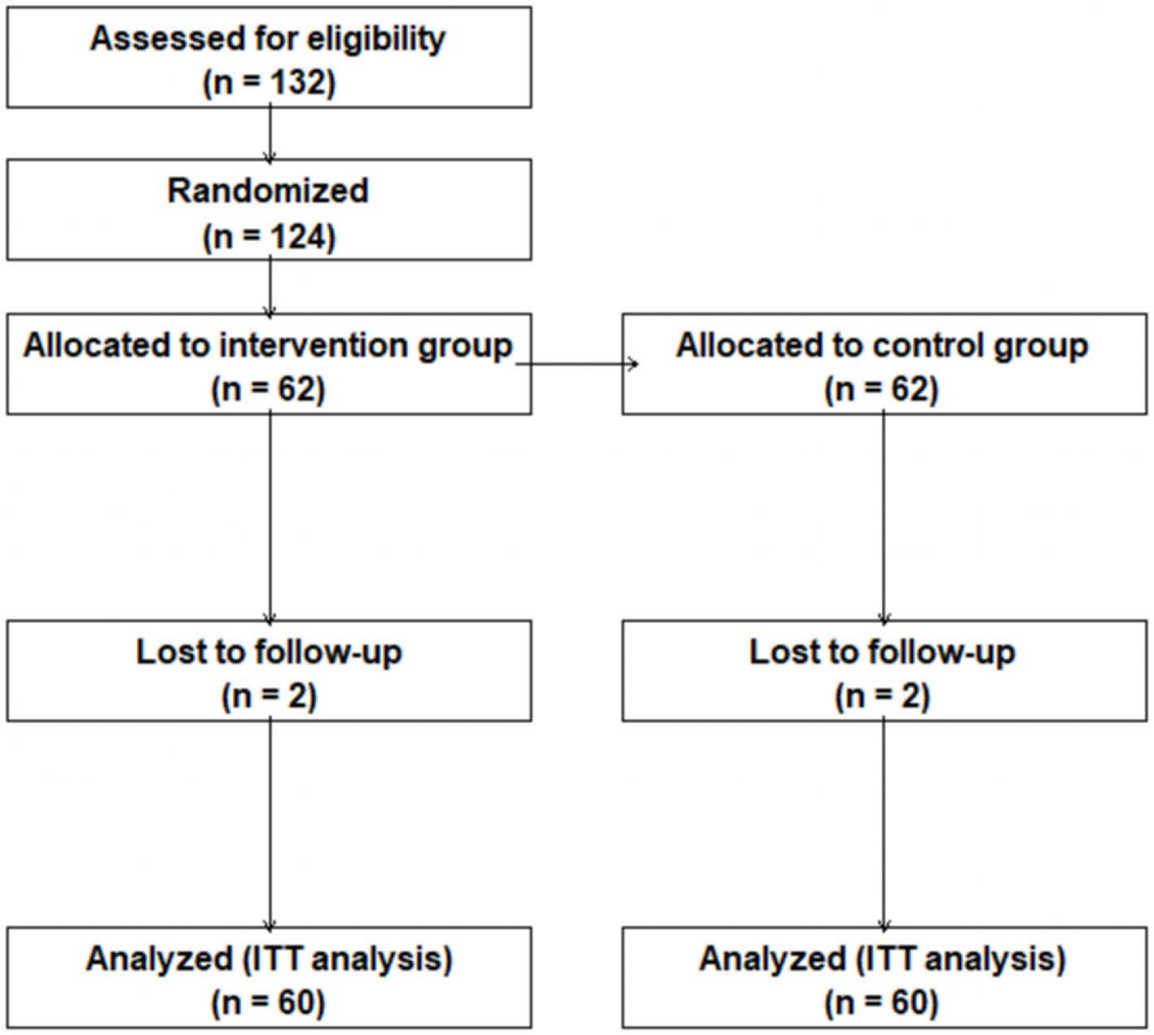
Participant flow diagram (CONSORT).

This study was reviewed and approved by the Ethics Committee of Tianjin Normal University (No. 2024081101). All procedures were conducted in accordance with the ethical standards for psychological and educational research involving children, and informed written consent was obtained from the legal guardians of all participating children prior to the study initiation. A CONSORT flow diagram was used to show participant enrollment, randomization, attrition and analysis (Figure 1).

### 2.2 Intervention

This study was conducted from September 2024 to December 2024 in public kindergartens in Tianjin. The baseline data of all participants were collected in the first week of September 2024, and the post-intervention follow-up data were collected in the last week of December 2024 after the 12-week intervention.

#### 2.2.1 Experimental group: traditional culture picture book intervention

*Intervention duration and frequency*: the intervention lasted 12 weeks, with two sessions per week and 45 min per session. A total of 24 sessions were conducted. All sessions were arranged from 10:00 to 10:45 in the morning, within regular kindergarten class hours without extra teaching time.

*Picture book selection criteria*: (1) the content reflected the core values of traditional Chinese culture and covered traditional virtues such as filial piety, loyalty, propriety, righteousness, integrity, shame, benevolence and kindness. Classic stories included Kong Rong Sharing Pears, Huang Xiang Warming the Quilt, and Sima Guang Breaking the Vat. (2) The pictures were vivid with soft colors, and the text was simple and understandable, which was suitable for the cognitive level of children aged 4 to 6 years. (3) Clear emotional scenes were included, such as joy, grievance, anger and kindness, to help children perceive and understand different emotions. (4) All books were reviewed by an expert team composed of three professors in preschool education and two senior kindergarten teachers to ensure cultural accuracy and educational appropriateness. A total of 12 core picture books were selected and rotated weekly.

*Intervention procedure*: (1) shared picture book reading (15 min): Teachers read the stories in an expressive way, combining body language and facial mimicry to enhance appeal and guide children to observe the emotional expressions of characters in the pictures. (2) Emotional discussion (10 min): Teachers raised questions around the emotional scenes in the picture book stories, encouraging children to express their views boldly. (3) Scenario simulation (15 min): Teachers selected key plots from the picture books, divided children into groups to conduct role-play, and guided them to practice emotional expression and regulation strategies. (4) Summary and extension (5 min): Teachers sorted out the cultural connotations and emotional regulation methods in the picture books, guiding children to apply them in daily life scenarios.

*Quality control*: teachers received unified training before the intervention to clarify intervention goals, procedures, and standards. An intervention record form was completed after each session to document children’s participation and task completion. A teaching research meeting was held once a week to solve problems in a timely manner. Members of the research team randomly checked 30% of all intervention sessions to ensure consistency and standardization.

#### 2.2.2 Control group: routine teaching

Children in the control group did not receive special picture book intervention. They participated in routine activities according to the original kindergarten teaching plan, including courses in language, science, art, health, and society. The total activity duration, teacher guidance time and teacher-student interaction frequency were fully matched between the two groups. To ensure comparability, irrelevant variables such as educational environment, teacher allocation, and daily activity duration were kept consistent between the two groups. The only difference was the intervention content. No active control group was set because routine teaching is the most consistent with real preschool education scenarios, ensuring the ecological validity of the study.

### 2.3 Observational indicators and evaluation methods

The primary outcomes were usage rate of emotion regulation strategies and sustained attention duration; secondary outcomes were other emotional regulation and cognitive function indicators.

#### 2.3.1 Indicators of emotion regulation ability

All assessment tools were validated for children aged 4–6 years with reported reliability, validity, norm references and inter-rater reliability.

##### 2.3.1.1 Accuracy of emotion recognition

Measured by the Preschool Children’s Emotional Facial Expression Scale (reliability coefficient = 0.86, validity = 0.79). It includes six basic emotions: happiness, sadness, anger, fear, surprise, and disgust. Five images are used for each emotion, with a total of 30 images. The images are shown to children in random order. Children are asked to identify the emotion verbally. The accuracy rate is calculated as the percentage of correctly identified images over the total number of images. The score ranges from 0 to 100%. A higher score indicates stronger emotion recognition ability. Inter-rater reliability = 0.88.

##### 2.3.1.2 Normative score of emotional expression

Measured by a situational test compiled with reference to the Preschool Children’s Emotional Expression Assessment Manual (validity = 0.76). Six daily scenarios are designed, such as receiving a gift, being misunderstood by peers, and having toys taken away. Children express their emotions through words, facial expressions, and behaviors. Two trained evaluators score each child independently using a unified 4-point scale. The average score is used as the final result. The grading criteria are as follows: 3.5–4.0 points for excellent, 2.5–3.4 points for good, 1.5–2.4 points for qualified, and 0–1.4 points for unqualified. A higher score represents more normative emotional expression. Inter-rater reliability = 0.85.

##### 2.3.1.3 Usage rate of emotion regulation strategies

Measured by the Preschool Children’s Emotion Regulation Strategy Observation Scale (reliability = 0.83). Natural observation is applied in this measurement. Observations are conducted for three consecutive days before and after the intervention. Each day includes two 1-h sessions, one in the morning and one in the afternoon. When children experience negative emotions such as crying, anger, or distress, the use of regulation strategies is recorded. These strategies include active communication, attention diversion, and self-comforting. The usage rate is the percentage of times regulation strategies are used relative to the total number of negative emotional events. A higher rate shows greater initiative in emotion regulation. Observation reliability = 0.82.

##### 2.3.1.4 Duration of negative emotions

During natural observation, the time from the onset to full recovery of each negative emotion episode is recorded. The average duration is calculated. A shorter duration indicates higher efficiency in emotion regulation.

#### 2.3.2 Cognitive function indicators

All assessment tools were validated for children aged 4–6 years with reported reliability, validity, norm references and standardized operation procedures.

##### 2.3.2.1 Sustained attention duration

Measured by the Preschool Children’s Digit Cancellation Test (reliability = 0.81), where a sheet with 500 randomly arranged digits from 0 to 9 is provided, and children are instructed to cancel target digits within 10 min. The longest duration of continuous correct cancellation without obvious distraction is recorded in minutes, and a longer duration indicates stronger sustained attention.

##### 2.3.2.2 Attention span

Measured by the Visual Search Test (reliability = 0.79), in which pictures with different numbers of geometric shapes including circles, squares and triangles are presented, and children are asked to count target shapes quickly within 30 s. The task starts with three shapes and gradually increases in difficulty, and the maximum number of shapes with three consecutive correct responses is taken as the measure of attention span.

##### 2.3.2.3 Reaction time in the Stroop task

Measured by the Preschool Children’s Color-Word Stroop Task (reliability = 0.84, validity = 0.78) with two designed conditions, congruent and incongruent; in the congruent condition, the font color matches the word meaning, while in the incongruent condition, the font color conflicts with the word meaning. Thirty stimuli are presented in random order for each condition, and the average reaction time for correct color judgments is recorded in milliseconds, with a shorter reaction time indicating stronger executive function, especially inhibitory control.

##### 2.3.2.4 Visuospatial cognitive score

Measured by the Preschool Children’s Block Design Test (reliability = 0.85), where a standardized block set is provided and children complete eight block-assembling tasks of increasing difficulty according to models or printed instructions. Each task is scored based on completion time and accuracy with a maximum score of 10 points, the total score is 80 points which is then converted to a 100-point scale, and a higher score represents stronger visuospatial cognitive ability.

##### 2.3.2.5 Working memory capacity

Measured by the Preschool Children’s Digit Span Backward Test (reliability = 0.82, validity = 0.75), in which the examiner orally presents a sequence of random digits starting with three digits, and children are required to repeat the digits in reverse order. The length of the digit sequence increases gradually, and the maximum digit length with two consecutive correct repetitions is used as the measure of working memory capacity.

All indicators are assessed once at pre-intervention baseline and once at post-intervention after 12 weeks; all assessors are trained with standardized protocols before evaluation, a blind assessment design is used in which assessors are unaware of group assignments, and test–retest reliability is examined for 20% of the data with the reliability coefficient ranging from 0.85 to 0.92, which confirms the reliability of the assessment results.

### 2.4 Quality control

During the research design stage, expert consultation was used to verify the scientific validity and feasibility of the intervention program and evaluation indicators. Random grouping was adopted to reduce selection bias, and standardized inclusion and exclusion criteria were applied to ensure the homogeneity of participants. Privacy protection measures, such as data anonymization and encrypted storage of raw data, were implemented for the personal information of children involved in the study.

In the data collection stage, unified assessment tools and operational procedures were applied. All assessors received standardized training and were qualified before formal evaluation. Blinded assessment was used to minimize measurement bias. Data were checked and entered in a timely manner, and a double-data entry system was established to guarantee data accuracy.

In the statistical analysis stage, four children dropped out of the study, including two in the experimental group and two in the control group. Two children withdrew due to kindergarten transfer caused by family relocation or parental request, and the other two dropped out for kindergarten transfer. The dropout rate was 3.33%. Multiple imputation was used to handle missing data, and intention-to-treat analysis was applied to ensure the objectivity of results. The suitability of statistical methods was tested to confirm the reliability of analytical outcomes.

### 2.5 Statistical methods

Data processing and analysis were performed using SPSS 26.0 software. Normality tests and homogeneity tests of variance were conducted first. All measurement data were normally distributed and presented as mean ± standard deviation. Paired-sample *t*-tests were used for intra-group comparisons before and after the intervention. Independent-sample t-tests were adopted to compare baseline data and post-intervention data between groups. Repeated-measures analysis of variance was applied to analyze dynamic changes in indicators during the intervention. Pearson correlation analysis was used to examine the relationship between emotional regulation indicators and cognitive function indicators. Bonferroni correction was used for multiple testing. The significance level was set at *α* = 0.05 with a two-sided test, and *p* < 0.05 was considered statistically significant. Cohen’s d effect sizes and 95% confidence intervals (CI) were calculated (*d* = 0.2 small, *d* = 0.5 medium, *d* = 0.8 large).

## 3 Results

### 3.1 Baseline characteristics of participants

Before the intervention, homogeneity tests were conducted on demographic characteristics, basic health and developmental baseline indicators between the two groups. The results showed no statistically significant differences in all indicators between groups, indicating that the two groups of children had consistent baseline characteristics and good comparability (Table 1).

**Table 1:** Comparison of baseline characteristics between two groups of children.

Indicator	Experimental group (*n* = 60)	Control group (*n* = 60)	Statistic	*p*-value
Gender (male/female, *n*)	32/28	31/29	*χ*^2^ = 0.033	0.855
Age (years, mean ± SD)	5.23 ± 0.67	5.18 ± 0.72	*t* = 0.386	0.700
Age distribution (*n*, %)			*χ*^2^ = 0.198	0.906
4 years	21 (35.0)	23 (38.3)		
5 years	25 (41.7)	24 (40.0)		
6 years	14 (23.3)	13 (21.7)		
Height (cm, mean ± SD)	112.45 ± 6.32	111.87 ± 6.54	*t* = 0.482	0.631
Weight (kg, mean ± SD)	20.36 ± 3.15	20.12 ± 3.28	*t* = 0.405	0.686
Birth weight (kg, mean ± SD)	3.25 ± 0.42	3.21 ± 0.45	*t* = 0.501	0.617
Family residence (*n*, %)			*χ*^2^ = 0.275	0.871
Urban	48 (80.0)	46 (76.7)		
Township	12 (20.0)	14 (23.3)		
Daily outdoor activity time (h, mean ± SD)	1.52 ± 0.43	1.48 ± 0.45	*t* = 0.498	0.619
Daily sleep time (h, mean ± SD)	9.56 ± 0.62	9.48 ± 0.65	*t* = 0.643	0.521
Past medical history (yes/no, *n*)	3/57	2/58	*χ*^2^ = 0.207	0.649
Duration of kindergarten attendance (months, mean ± SD)	14.25 ± 5.36	13.87 ± 5.42	*t* = 0.392	0.696

### 3.2 Comparison of emotional regulation indicators between two groups after intervention

**Figure 2:**
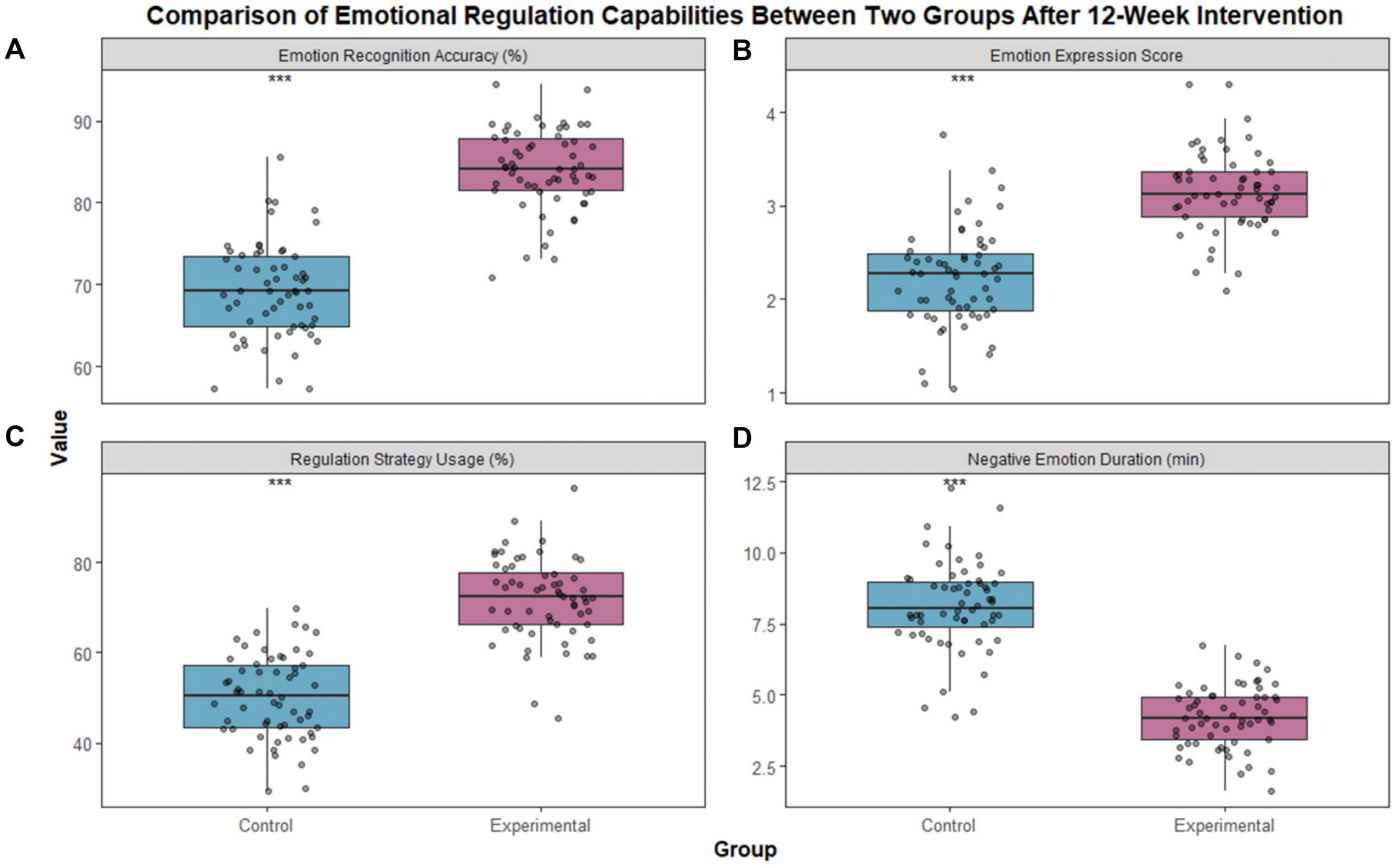
Comparison of emotional regulation indicators between two groups of children after intervention. (**A**) represents accuracy of emotion recognition, (**B**) represents score of emotional expression normality, (**C**) represents utilization rate of emotion regulation strategies, and (**D**) represents duration of negative emotions. ***p < 0.001 compared with the control group.

Repeated-measures ANOVA showed significant main effects of time, group, and time × group interaction (all *p* < 0.001). After 12 weeks of intervention, children in the experimental group showed significant advantages in all four core indicators of emotional regulation. The differences between the experimental group and the control group were statistically significant (Table 2; Figure 2). All Cohen’s d values were 1.23–2.15 (95% CI did not include 0), indicating large effects.

**Table 2:** Comparison of emotional regulation indicators between two groups of children after intervention (mean ± SD).

Indicator	Group	Baseline	Post-intervention	Within-group change	*t*-value	*p*-value	Between-group *t*-value	*p*-value	Cohen’s d (95%CI)
Accuracy of emotion recognition (%)	Experimental	65.31 ± 7.24	84.72 ± 5.36	19.41 ± 6.18	14.52	<0.001	12.894	<0.001	1.86 (1.42, 2.30)
	Control	64.87 ± 7.51	68.54 ± 6.72	3.67 ± 4.25	4.01	<0.001	–	–	–
Normative score of emotional expression	Experimental	1.85 ± 0.52	3.12 ± 0.45	1.27 ± 0.48	12.36	<0.001	9.673	<0.001	1.53 (1.12, 1.94)
	Control	1.81 ± 0.54	2.21 ± 0.53	0.40 ± 0.36	5.12	<0.001	–	–	–
Usage rate of emotion regulation strategies (%)	Experimental	42.16 ± 9.35	72.35 ± 8.41	30.19 ± 7.62	17.85	<0.001	11.235	<0.001	2.15 (1.68, 2.62)
	Control	41.82 ± 9.61	51.68 ± 9.25	9.86 ± 5.73	7.45	<0.001	–	–	–
Duration of negative emotions (minutes)	Experimental	7.95 ± 1.63	4.32 ± 1.25	−3.63 ± 1.41	−12.78	<0.001	−12.017	<0.001	1.23 (0.85, 1.61)
	Control	7.88 ± 1.67	7.86 ± 1.58	−0.02 ± 0.85	−0.15	0.881	–	–	–

### 3.3 Comparison of cognitive function indicators between two groups after intervention

**Figure 3:**
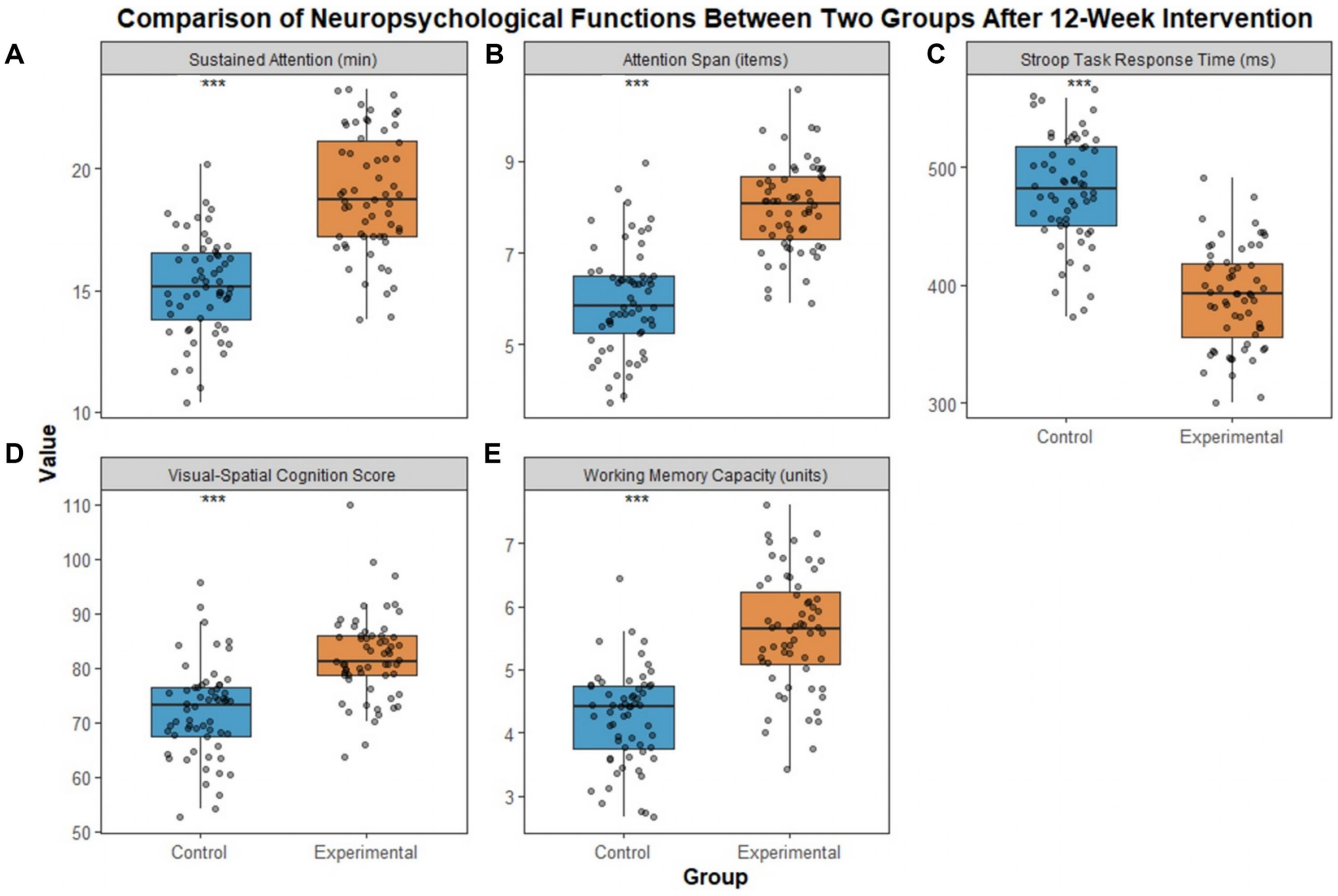
Comparison of neuropsychological indicators between two groups of children after intervention. (**A**) represents duration of sustained attention, (**B**) represents attention span, (**C**) represents reaction time in the Stroop task, (**D**) represents visuospatial cognition score, and (**E**) represents working memory capacity. ***p < 0.001 compared with the control group.

Repeated-measures ANOVA showed significant main effects of time, group, and time × group interaction (all *p* < 0.001). After 12 weeks of intervention, cognitive functions of children in the experimental group were significantly improved. Sustained attention duration, attention span, visuospatial cognition score, and working memory capacity were all significantly higher in the experimental group than in the control group. The reaction time in the Stroop task was significantly shorter in the experimental group. All five indicators showed statistically significant differences (Table 3; Figure 3). All Cohen’s d values were 1.16–1.98 (95% CI did not include 0), indicating large effects.

**Table 3:** Comparison of cognitive function indicators between two groups after intervention (mean ± SD).

Indicator	Group	Baseline	Post-intervention	Within-group change	*t*-value	*p*-value	Between-group *t*-value	*p*-value	Cohen’s d (95%CI)
Sustained attention duration (minutes)	Experimental	12.35 ± 2.42	18.76 ± 2.15	6.41 ± 2.36	12.65	<0.001	7.342	<0.001	1.67 (1.25, 2.09)
	Control	12.28 ± 2.47	15.42 ± 2.43	3.14 ± 1.85	7.26	<0.001	—	—	—
Attention span (items)	Experimental	5.24 ± 1.13	7.89 ± 1.02	2.65 ± 0.94	12.87	<0.001	8.015	<0.001	1.78 (1.35, 2.21)
	Control	5.19 ± 1.16	6.12 ± 1.15	0.93 ± 0.72	6.35	<0.001	—	—	—
Reaction time in the Stroop task (ms)	Experimental	465.72 ± 51.36	386.54 ± 42.37	−79.18 ± 26.54	−9.65	<0.001	−9.876	<0.001	1.98 (1.53, 2.43)
	Control	462.38 ± 52.15	478.62 ± 48.59	16.24 ± 18.36	3.56	<0.01	—	—	—
Visuospatial cognitive score (points)	Experimental	62.18 ± 8.35	82.45 ± 6.78	20.27 ± 7.36	13.24	<0.001	8.923	<0.001	1.82 (1.38, 2.26)
	Control	61.92 ± 8.42	70.32 ± 7.54	8.40 ± 5.27	7.85	<0.001	—	—	—
Working memory capacity (units)	Experimental	3.52 ± 0.84	5.68 ± 0.87	2.16 ± 0.75	13.68	<0.001	8.364	<0.001	1.62 (1.20, 2.04)
	Control	3.48 ± 0.86	4.21 ± 0.92	0.73 ± 0.61	6.12	<0.001	—	—	—

### 3.4 Correlation between emotional regulation and neuropsychological indicators in preschool children

**Figure 4:**
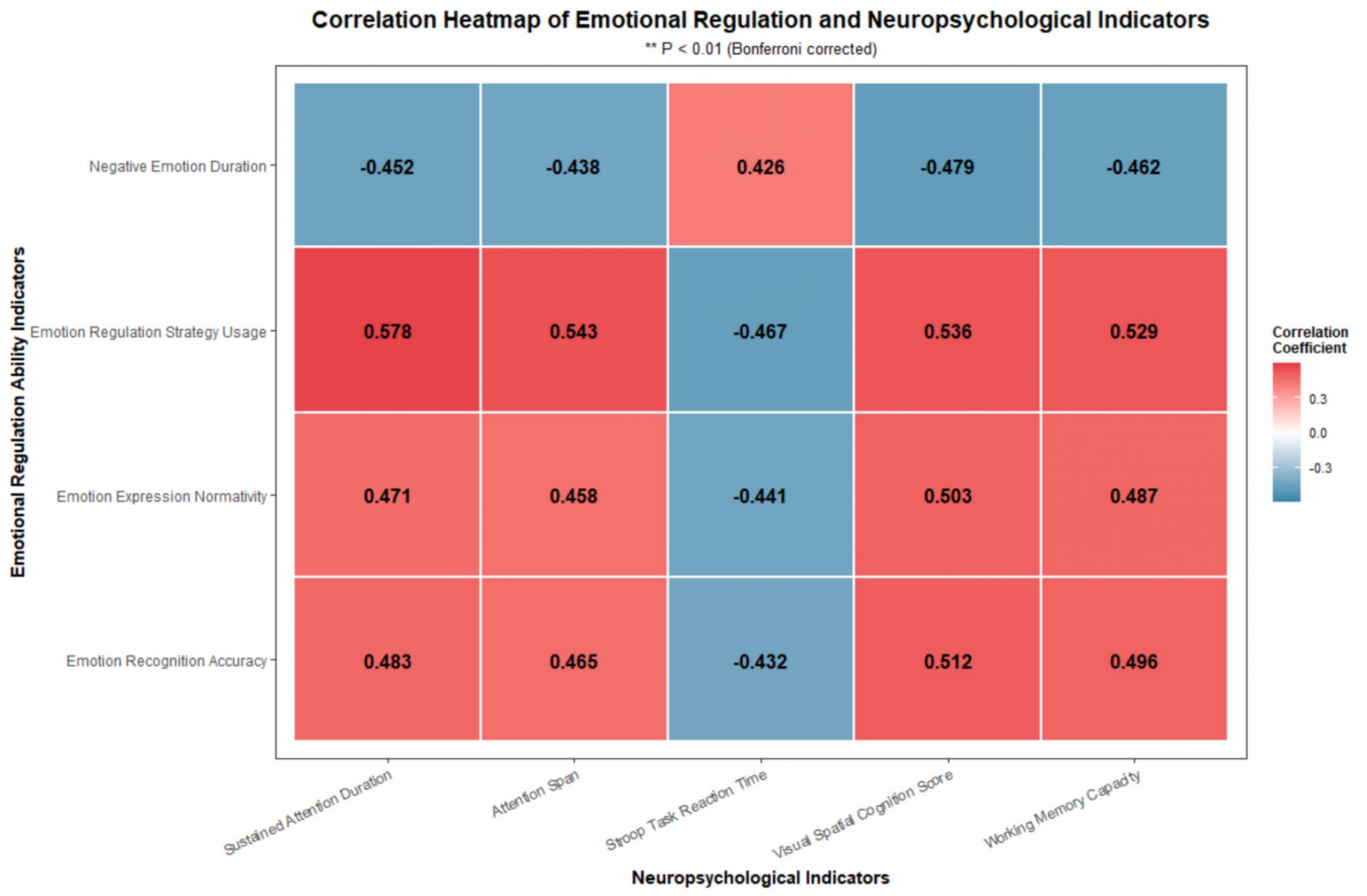
Correlation heatmap of emotional regulation ability and neuropsychological indicators. Darker color indicates stronger correlation. Red represents positive correlation, and blue represents negative correlation. **p < 0.01.

Pearson correlation analysis showed that the four indicators of emotional regulation were significantly correlated with the core indicators of cognitive functions (*r* = 0.426–0.578, *p* < 0.01) (Table 4; Figure 4). Among them, the use of emotional regulation strategies had the strongest positive correlation with sustained attention duration. In contrast, the duration of negative emotion was positively correlated with response time in the Stroop task (*r* = 0.426, *p* < 0.01). These results only indicate a significant association, not a causal or directional relationship.

**Table 4:** Correlation analysis between emotional regulation and neuropsychological indicators (*r* values).

Emotional regulation indicators	Sustained attention duration	Attention span	Stroop task response time	Visuospatial cognition score	Working memory capacity
Emotional recognition accuracy rate	0.483**	0.465**	−0.432**	0.512**	0.496**
Emotional expression normativity score	0.471**	0.458**	−0.441**	0.503**	0.487**
Usage rate of emotional regulation strategies	0.578**	0.543**	−0.467**	0.536**	0.529**
Duration of negative emotions	−0.452**	−0.438**	0.426**	−0.479**	−0.462**

**p < 0.01, remains statistically significant after Bonferroni correction for multiple testing.

## 4 Discussion

Preschool children are in a critical period for emotional regulation and cognitive function development. Their brain development is not yet mature, and their emotional control and cognitive abilities need to be improved (18). As a core dimension of social development in preschool children, the quality of emotional regulation directly affects children’s social adaptation and mental health (19).

In this study, after intervention with traditional Chinese picture books, children in the experimental group showed an emotional recognition accuracy rate of 84.72%, which was significantly higher than the 68.54% observed in the control group. Similar advantages were found in the normativity score of emotional expression and the usage rate of regulation strategies. Meanwhile, the duration of negative emotions was significantly shortened. These results are consistent with previous findings that culturally immersive education improves children’s emotional cognition (20). The underlying mechanism may lie in the unique educational value of traditional picture books. On the one hand, cultural concepts such as benevolence, kindness, and perspective-taking are highly consistent with cognitive reappraisal strategies in emotional regulation theory. They help children build a positive framework for emotional understanding (21). On the other hand, concrete story scenes and localized cultural symbols, such as courtesy in Kong Rong Shares Pears and gratitude in Huang Xiang Warms the Bed, easily arouse children’s emotional resonance. Children thus learn to recognize and appropriately express emotions through immersive experience (22).

Cognitive function development provides an important foundation for improving cognitive abilities in preschool children. Core indicators including attention, working memory, and executive function are closely related to the development of the prefrontal cortex (23, 24). Results of this study show that children in the experimental group performed significantly better than the control group in sustained attention duration, attention span, visuospatial cognition score, and working memory capacity. Their response time in the Stroop task was also significantly shorter. These findings suggest that traditional picture book intervention effectively promotes the comprehensive development of cognitive functions in children. This discovery expands the scope of existing research. Previous studies mostly focused on the effects of picture books on language ability or aesthetic literacy, while the present study confirms their positive role in cognitive functions (25). From a neural mechanism perspective, the intervention follows a closed-loop process: shared reading, discussion, situational simulation, and extended application. This process requires children to focus on understanding the story, which enhances sustained attention and attention span. It also encourages them to use memory resources to integrate plot logic, thus strengthening working memory capacity. In role-play activities, children further practice visuospatial cognition and problem-solving skills (26). The logical narrative structure of picture books and dialectical thinking in traditional culture contribute to improved inhibitory control in the Stroop task (27).

Correlation analysis in this study reveals that all indicators of emotional regulation are significantly associated with cognitive function indicators in preschool children (*r* = 0.426–0.578, *p* < 0.01). The strongest correlation appears between the usage rate of emotional regulation strategies and sustained attention duration. This correlation does not imply causation or directional influence. The prefrontal cortex is related to both emotional regulation and higher cognitive functions, which may explain the observed association (28, 29). Specifically, improved sustained attention helps children detect emotional cues more sensitively, supporting better emotional recognition and expression. Increased working memory capacity allows children to quickly retrieve learned regulation strategies during emotional arousal, thereby improving emotional regulation efficiency (30). In turn, better emotional regulation reduces the occupation of cognitive resources by negative emotions. This creates favorable conditions for cognitive function performance and forms a positive cycle between cognition and emotion (31, 32).

This study has three main innovations. First, it establishes a three-in-one intervention model integrating traditional culture inheritance, emotional education, and cognitive function development. This model breaks through the limitations of traditional emotional education, such as monotonous forms and weak cultural roots, and provides new ideas for developing localized intervention programs. Second, it examines both emotional regulation and cognitive function development. Through multi-index quantitative analysis, it comprehensively reveals the comprehensive effects of traditional picture book intervention and addresses the lack of multi-dimensional research. Third, the intervention program is highly practical. The 12-week intervention, twice weekly and 45 min each session, matches regular kindergarten teaching schedules. Picture books are easily accessible and cost-effective, making the program suitable for widespread use in kindergartens.

This study also has several limitations. First, the sample was limited to public kindergartens in Tianjin using convenience sampling, which restricts regional representativeness and generalizability. Second, teachers were aware of group assignments, which may lead to expectation bias and differential interaction affecting the results. Third, no active control group was set, so it is impossible to fully distinguish whether the intervention effect comes from traditional culture content, structured activities or their combination. Fourth, the long-term stability of intervention effects remains unclear; follow-up studies are needed to examine effects 6 to 12 months after intervention. Fifth, potential confounding factors such as children’s temperament, parenting styles and family environment were not controlled. Sixth, this study is a small-sample cluster RCT with only four classes included. Although cluster adjustment was omitted due to the very low ICC, this small-sample cluster design still carries potential statistical test bias, which is a methodological limitation that needs to be addressed in future research by increasing the number of clusters and strictly performing cluster effect adjustment. Future studies may expand the sample, adopt third-party evaluation, set active control groups, conduct long-term follow-ups, increase the number of clusters, perform strict cluster effect adjustment, and perform subgroup analyses to address these limitations.

## 5 Conclusion

This cluster randomized controlled trial investigated 120 4–6-year-old preschool children in Tianjin, who were divided into an experimental group (12-week traditional culture picture book intervention) and a control group (routine teaching). Both groups had equal class time and teacher attention. The results showed that the experimental group performed significantly better than the control group in all emotional regulation and cognitive function indicators (all *p* < 0.001). Repeated-measures ANOVA confirmed significant time, group and interaction effects, with large Cohen’s d effect sizes and 95% CIs. Pearson correlation analysis revealed a significant association between emotional regulation ability and cognitive functions (*r* = 0.426–0.578, *p* < 0.01).

This study confirms that traditional culture picture book intervention can effectively improve preschool children’s emotional regulation ability and promote cognitive function development, and this intervention model is highly operable and compatible with routine kindergarten teaching, which can serve as an effective localized educational carrier for preschool education. The study is limited by its regional sample, unclear long-term intervention effects and unexamined influencing factors such as children’s temperament, which need to be addressed in future follow-up and expanded research. In general, traditional culture picture book intervention has important practical value for the comprehensive development of preschool children, and it is worthwhile to further explore and popularize its educational application in preschool education.

## Data Availability

The raw data supporting the conclusions of this article will be made available by the authors, without undue reservation.
